# Overfishing and sea warming drive the collapse of *Paracentrotus lividus*

**DOI:** 10.1038/s41598-025-02642-3

**Published:** 2025-05-28

**Authors:** Andrea Toso, Francesca Necci, Alessandra Martines, Roberta Lacorte, Yann Toso, Paola Gianguzza, Alan Deidun, Nicola Ungaro, Gaetano Costantino, Marianna Caforio, Cosimo Gaspare Giannuzzi, Francesco Marco D’Onghia, Giuseppe Strippoli, Enrico Barbone, Giacomo Milisenda, Stefano Piraino

**Affiliations:** 1 Department of Biological and Environmental Sciences and Technologies (DiSTeBA), University of Salento, Lecce, Italy; 2 National Biodiversity Future Center (NBFC), 90133 Palermo, Italy; 3 Consorzio Nazionale Interuniversitario per le Scienze del Mare (CoNISMa), Roma, Italy; 4 Università degli Studi di Palermo, via Archirafi 18, 90123 Palermo, Italy; 5 Università di Pisa, via Derna 1, 56126 Pisa, Italy; 6 Department of Integrative Marine Ecology, Stazione Zoologica Anton Dohrn, Genova Marine Centre, 16126 Genova, Italy; 7 Oceanography Malta Research Group (OMRG), Department of Geosciences, University of Malta, Msida, 2080 Malta; 8 Apulian Regional Agency for the Environmental Prevention and Protection, 70126 Bari, Italy; 9 Department of Integrative Marine Ecology, Stazione Zoologica Anton Dohrn, Sicily Marine Centre, Palermo, PA Italy

**Keywords:** Biodiversity loss, Rocky bottoms, Sea urchin harvesting, Meta-analysis, Climate change, Ecology, Environmental sciences

## Abstract

As a seafood delicacy, the purple sea urchin *Paracentrotus lividus* is the most exploited echinoid species in the Mediterranean Sea, with a significant increase in total catches being reported over the last 2 decades. This work presents: (i) the findings from two demographic monitoring campaigns, conducted on *P. lividus* populations in summer 2023, at shallow depths within the coastal areas of Sicily and Apulia; (ii) a meta-analysis of the *P. lividus* density in the Mediterranean Sea over the last 30 years. In both sampled areas, the recorded individual density was lower than ever, with an average value of 0.2 ind/m^2^. No significant difference in *P. lividus* density was detected between protected and non-protected areas. The observed rarity of the edible sea urchin is corroborated by a meta-regression analysis of data from 44 published articles over the period 1990–2020, showing that the decline of *P. lividus* populations in the Mediterranean Sea began in 2003, coincident with the occurrence of a pan-European heat wave and with the anomalous warming of the entire Mediterranean Sea.

**Supplementary Information:**

The online version contains supplementary material available at 10.1038/s41598-025-02642-3.

## Introduction

Fishing (commercial and recreational) is one of the major anthropogenic pressures on marine ecosystems, with the consequence that most commercial fish stocks are fully exploited, overexploited or depleted^1^.

As an indirect consequence, this overfishing causes significant problems for wildlife by leading to habitat loss, habitat fragmentation, and increased human-wildlife conflict^2–4^. In the last decades, also due the increasing depletion of fish stocks, a concurrent fishery of invertebrates has constantly increased^5^. Due to their highly palatable roe (i.e., the echinoid ovaries or testes), the demand for sea urchins as seafood has risen significantly across various regions of the world, including Europe, Asia, and America. Over the last 2 decades, this increased demand conversely led to a decline in the natural populations of the harvested species, whose natural reproductive potential can be inadequate to match the market demand^6–9^. Technological advancements, e.g. for SCUBA diving, have facilitated increased harvesting of the species, further straining the exploited populations.

The purple sea urchin *Paracentrotus lividus* (Lamarck, 1816), is the most exploited echinoid species in the Mediterranean Sea, with a reported increase in the total catches of the species from 66 t in 2013 to 387 t in 2019 in the Mediterranean basin^10^. Distributed in the entire Mediterranean Sea, as well as in North-Eastern Atlantic coastal waters stretching from Ireland to the coasts of Morocco, *P. lividus* inhabits shallow rocky bottoms colonized by photophilic algae and *Posidonia oceanica* meadows^11,12^. In spite of its omnivorous predatory potential, it mainly feeds on fleshy, erect algae and occasionally on bryozoans, hydrozoans and polychaetes^13^. *P. lividus* is an iteroparous, gonochoristic species, with several broadcast spawning events over the year^14–16^. Fertilized oocytes develop into planktotrophic, echinopluteus larvae with a high dispersal potential^17,18^.

In European seas, the presence and relative abundance of this species is linked to the occurrence of two priority habitats (as recognized within Annex I of the Habitats Directive—Dir 92/43/CEE), i.e., *Posidonia oceanica* meadows (code 1170) and shallow subtidal rocky reefs (code 1120). In the Mediterranean Sea, *P. lividus* acts as a key species in subtidal ecosystems by controlling benthic algal communities^19,20^. For this reason, *P. lividus* is also included in Annex V of the Habitats Directive, Annex III of the SPA/BIO Protocol to the Barcelona Convention and in Annex III of the Bern Convention, as a species whose exploitation should be regulated. Indeed, *P. lividus* is able to modify the primary production of rocky bottoms environments^13,16,21–23^. High densities of *P. lividus* (> 7 ind/m^2^) and *Arbacia lixula* (Linnaeus, 1758) can lead to the transformation of the marine environment they inhabit, simplifying it from a complex habitat, including the macro-algal forests formed by many species of the genus *Cystoseira*, to a barren benthic environment dominated only by encrusting Corallinaceae^16^. In situ experiments demonstrate that the removal of sea urchins in the Mediterranean Sea led to a reduction in the occurrence barren grounds, favoring the recovery of macroalgal stands^24,25^.

The maximum individual density attained by *P. lividus* can range between a few to a dozen specimens per m^2^, reaching a maximum value of 50–100 ind/m^2^ in Mediterranean shallow waters^16^.

Over the past years, high sea urchin densities (i.e. > 10 ind/m^2^) were reported across different Mediterranean countries, from Spain^26–28^ to France^29–31^, Italy^32–34^, Croatia^35^, Israel^36^, Türkiye^37^ and Egypt^38^.

As for most ectothermic organisms, the physiological fitness and geographical distribution of *P. lividus* are largely influenced by temperature; being tolerant to daily and seasonal fluctuations, in the Mediterranean Sea the optimal fertilization temperature is below 22 °C^39^, whereas prolonged exposure to high sea temperatures (e.g. > 30 °C, which represent end-of-summer sea temperatures in many eastern and central Mediterranean regions) can be responsible for the collapse of entire populations, as it happened in the Levantine sea^40^.

The density and size structure of *P. lividus* populations are also regulated by predation rates, especially by the fish species S*parus aurata* Linnaeus, 1758, *Diplodus sargus* (Linnaeus, 1758), *Diplodus vulgaris* (Geoffroy Saint-Hilaire, 1817), *Coris julis* (Linnaeus, 1758) and *Thalassoma pavo* (Linnaeus, 1758) and a few carnivore invertebrates, such as *Hermodice carunculata* (Pallas, 1766) and *Marthasterias glacialis* (Linnaeus, 1758)^16,41–46^. As a consequence, the density of sea urchins is often lower in the Marine Protected Areas (MPAs), where predatory fish are usually more abundant and larger with respect to those in non-protected areas^28^. In addition, the density of sea urchins in the Mediterranean Sea is also affected by uncontrolled exploitation from recreational and professional fisheries; in recent years, the increase in demand for sea urchins has caused a significant change in the size structure of populations, with the resulting decline of natural stocks and their reproductive potential^9,15,20,47–49^. As a direct consequence of the widespread, often illegal, over-harvesting of sea urchins, no differences between protected and unprotected zone sea urchin individual density values were observed in Sardinia^9^.

Indeed, sea urchin harvesting lacks a common regulatory framework across the Mediterranean Sea. Regulations vary significantly among country, with some of them having no specific laws governing the practice^47,50^ as in the case of Algeria, Libya, Egypt, Syria, and Lebanon. For other Mediterranean countries, different levels of restrictions can be found. Since 2022, Türkiye totally banned the fishery of *P. lividus* in the Marmara Sea, due to the depletion of the species along coastal areas. In Greece, a sea urchin fishing law has been introduced in 2014, limiting the fishing to six months per year (January, February, March, July, August and December) and the daily catch per boat to 600 sea urchins, with a minimum harvestable size diameter of 50 mm^51^. In Italy, sea urchin fishing has regulated since 1995 through a national ministry decree (MIPAAF D.M. 12/1/1995), banning sea urchin fishing in Italy for two months each year (May and June) and setting for the remaining months (from July to April) a daily fishing quota of 1000 individuals for professional fishermen, reduced to 50 specimens for recreational fishers. The minimal harvestable size is set at a diameter of 7 cm, including spines. In addition, two Italian regional governments (Sardinia and Apulia) decided to increase the level of regulation through regional laws. In the Sardinia region, sea urchin harvesting is now allowed from November to April, with a minimum sea urchin harvestable size (test diameter of at least 50 mm) and with a daily catch quota of 1500–3000 sea urchins^20^. In the Apulia region, the regional law No. 6 of April 18, 2023 imposed a three-year prohibition of the harvesting of *P. lividus* along the Apulian coast. Nonetheless, the trade of *P. lividus* harvested from other regions or from other countries was left in an unregulated state.

In this scenario, in order to gather updated information on the distribution and health status of different edible sea urchin populations, a stock assessment of the *P. lividus* populations was carried out in summer 2023, along approximately 2500 km of the Italian coastline, spread over two separate Italian regions, i.e., Apulia and Sicily, both within protected and within non-protected marine coastal areas.

By comparing the densities of different size classes and the total density of urchin specimens inside and outside MPAs, we evaluated differences in the structure of *P. lividus* populations due to the top-down control exerted by natural predation (inside MPAs) or by human harvesting (non-protected areas). Additionally, we carried out an extensive review of existing sea urchin density studies conducted throughout the Mediterranean Sea from 1990 to 2020, to identify and analyze potential trends in *P. lividus* populations over the past 30 years.

## Results

### Survey: Apulia

**Fig. 1:**
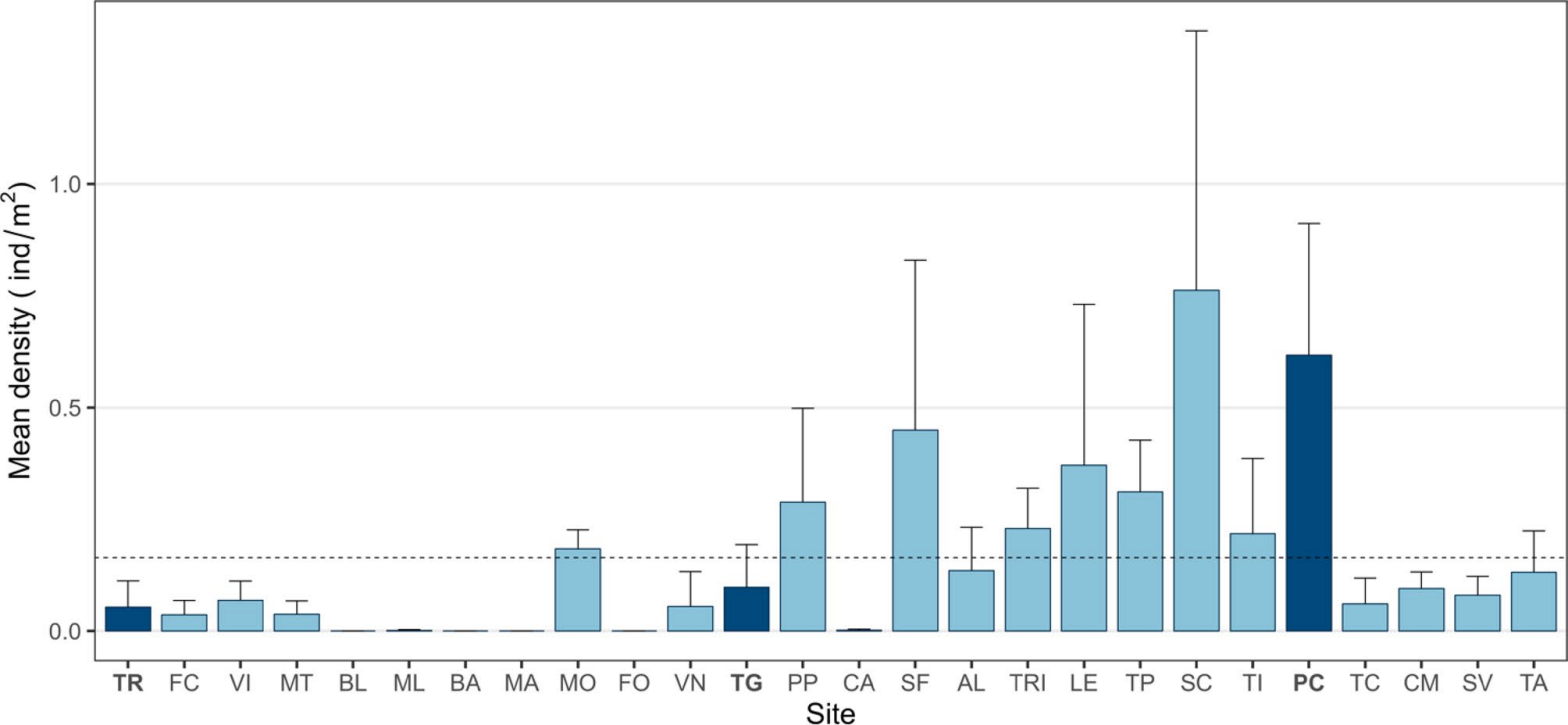
Mean density ind/m^2^ + S.D. of *Paracentrotus lividus* in the 26 sites along the coast of Apulia. For each site, three replicates were conducted. Dashed line is the mean value calculated for all the sites. MPAs are shown in dark blue, and non-protected areas in light blue. TR, Tremiti Islands (MPA); FC, Varano; VI, Vieste; MT, Mattinata; BL, Barletta; ML, Molfetta; BA, Bari; MA, Mola di Bari; MO, Monopoli; FO, Forcatella; VN, Villanova; TG, Torre Guaceto (MPA); PP, Punta Penne; CA, Casalabate; SF, San Foca; AL, Alimini; TRI, Tricase Porto; LE, Santa Maria di Leuca; TP, Torre Pali; SC, Santa Caterina; TI, Torre Inserraglio; PC, Porto Cesareo (MPA); TC, Torre Colimena; CM, Campomarino; SV, San Vito; TA, Taranto.

In summer 2023, a total of 41,766 m^2^ of seabed, spread across 26 sites, was surveyed along the Apulian coastline, resulting in a total count of 5882 *P. lividus* individuals of different sizes. The average individual density of sea urchin specimens was 0.2 ± 0.2 ind/m^2^ (Mean ± S.D.) and varied between 0 and 0.8 ± 0.6 ind/m^2^ (Fig. 1). The density varied considerably across sites. Notably, no sea urchins were observed at the Barletta, Bari, Mola di Bari, and Forcatella sites, while only two and three sea urchins were recorded at the Molfetta and Casalabate sites, respectively.

**Fig. 2:**
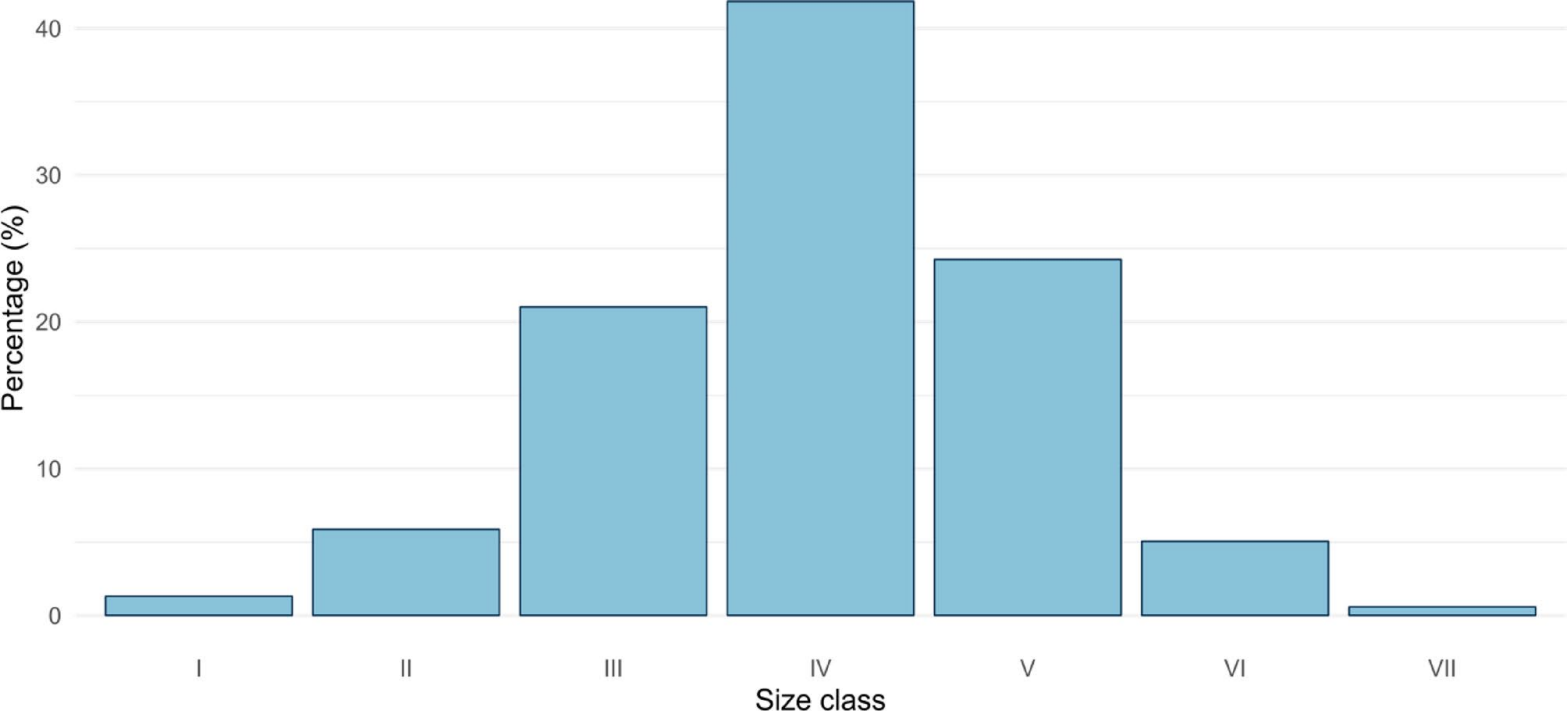
Size frequency histogram of the *P. lividus* in Apulia. I (0–10 mm); II (10–20 mm); III (20–30 mm); IV (30–40 mm); V (40–50 mm); VI (50–60 mm); VII (> 60 mm).

The mean size of *P. lividus* recorded along the Apulian coast was that of 34 ± 10 mm (Mean ± S.D.), as recorded from 5882 sampled specimens. The frequency distribution (Fig. 2) shows that size class IV (30–40 mm) was the most widespread, responsible for 41.8% of the total frequency, followed by size classes V (40–50 mm) and III (20–30 mm) with 24.2% and 21% of the total frequency, respectively. Sea urchins of commercial size (test diameter without spines equal or above 50 mm) constituted just 6% of the total sea urchin population surveyed in Apulia. 80% of the total recorded sea urchins were found in the depth range 0–5 m. The PERMANOVA analysis showed no statistical difference in size classes’ frequency between MPAs and non-protected areas (Pseudo-F = 0.41; *p* = 0.7); conversely, the same analysis revealed significant differences among sites with the same level of protection (Pseudo-F = 4.50; *p* = 0.0001). Similar results emerged from the analysis of total individual density inside MPAs and within non-protected areas (Pseudo-F = 0.08; *p* = 0.8), and for sites (Pseudo-F = 3.80; *p* = 0.0007).

### Survey: Sicily

**Fig. 3:**
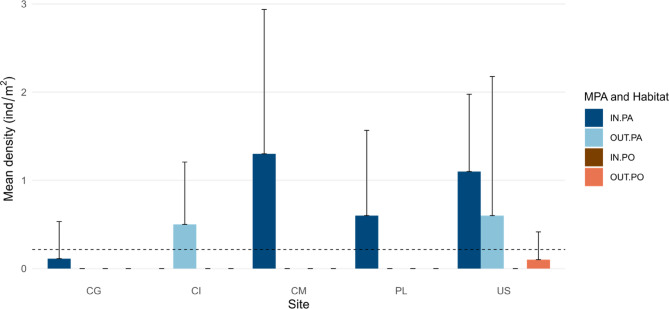
Mean density ind/m^2^ ± S.D. of *P. lividus* in the 5 sites along the coast of Sicily (CG, Capo Gallo; CI, Ciclopi Island; CM, Capo Milazzo; PL, Plemmirio; US, Ustica) in two habitats (PA, Algae photophilic; PO, *Posidonia oceanica*).

In the Sicily region, a total of 200 m^2^ of seabed, spread across 5 sites, was surveyed in summer 2023, resulting in a total count of 44 *P. lividus* individuals. The average individual density of *P. lividus* was that 0.2 ± 0.4 ind/m^2^ (Mean ± S.D.), varying between 0 and 1.3 ± 1.6 ind/m^2^ (Fig. 3). The two habitats investigated yielded a total of 43 individuals of *P. lividus* from the photophilic algae assemblages and just one individual from the *Posidonia oceanica* meadows. The mean size of *P. lividus* recorded along the Sicilian coast was that of 46 ± 22 mm (here including spine length measurement), based on 44 sampled specimens. The PERMANOVA analysis showed no statistical differences in size classes’ frequency values between MPAs (ns) and unprotected areas (Pseudo-F = 2.02; *p* = 0.19); also, the same analysis revealed no difference among sites at the same protection level (Pseudo-F = 1.41; *p* = 0.1). The results from the analysis of total frequency for MPAs and non-protected areas were not significant (Pseudo-F = 2.07; *p* = 0.2) but were significant for sites of the same protection category (Pseudo-F = 2.54; *p* = 0.03).

### Historical analysis (1990–2020) of *P. lividus* density across the Mediterranean Sea

**Fig. 4:**
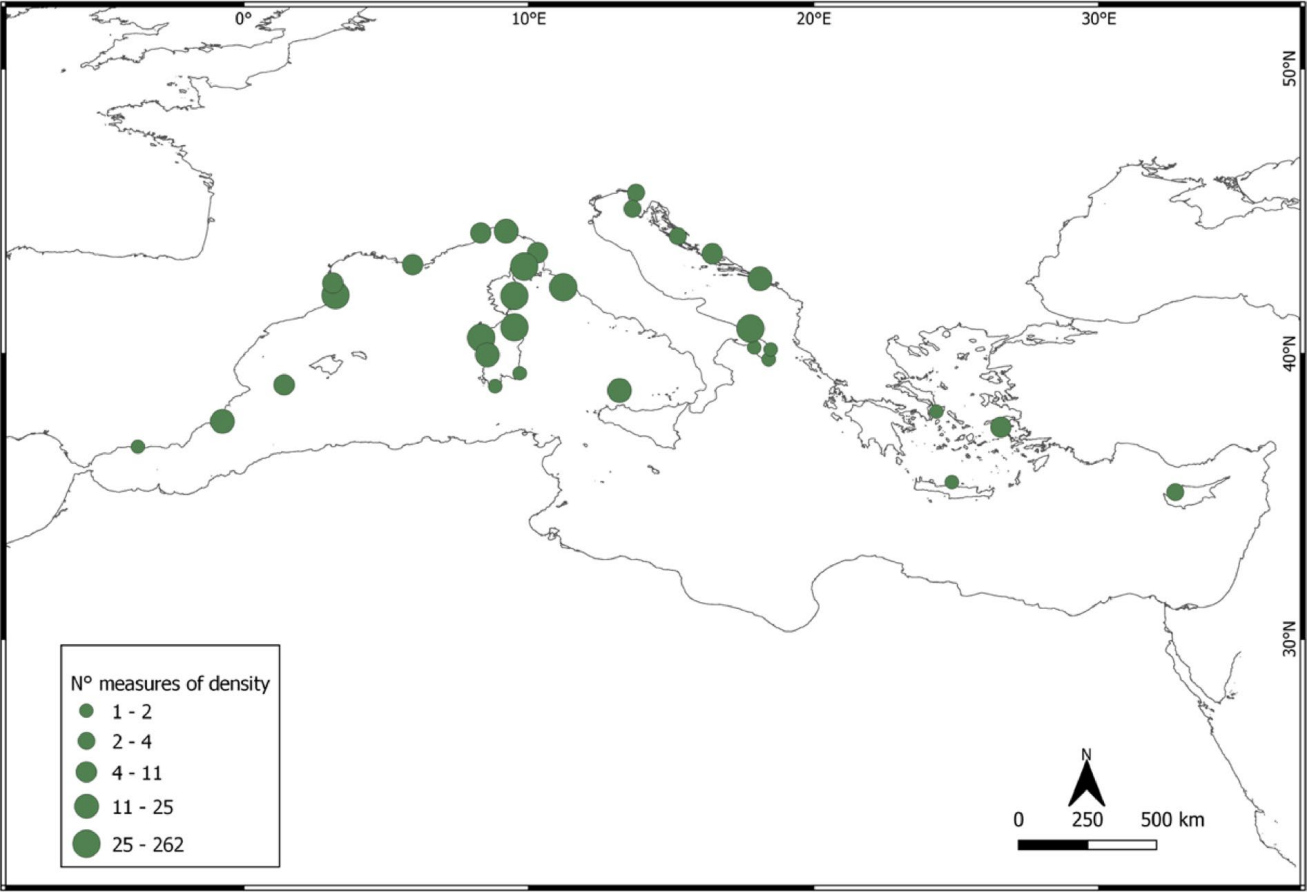
Map of Mediterranean localities where *Paracentrotus lividus* density data have been collected over the past 30 years. The size of each green circle indicates the number of density measurements recorded at that locality. Nearby localities are spaced at a minimum distance of 50 km.

**Fig. 5:**
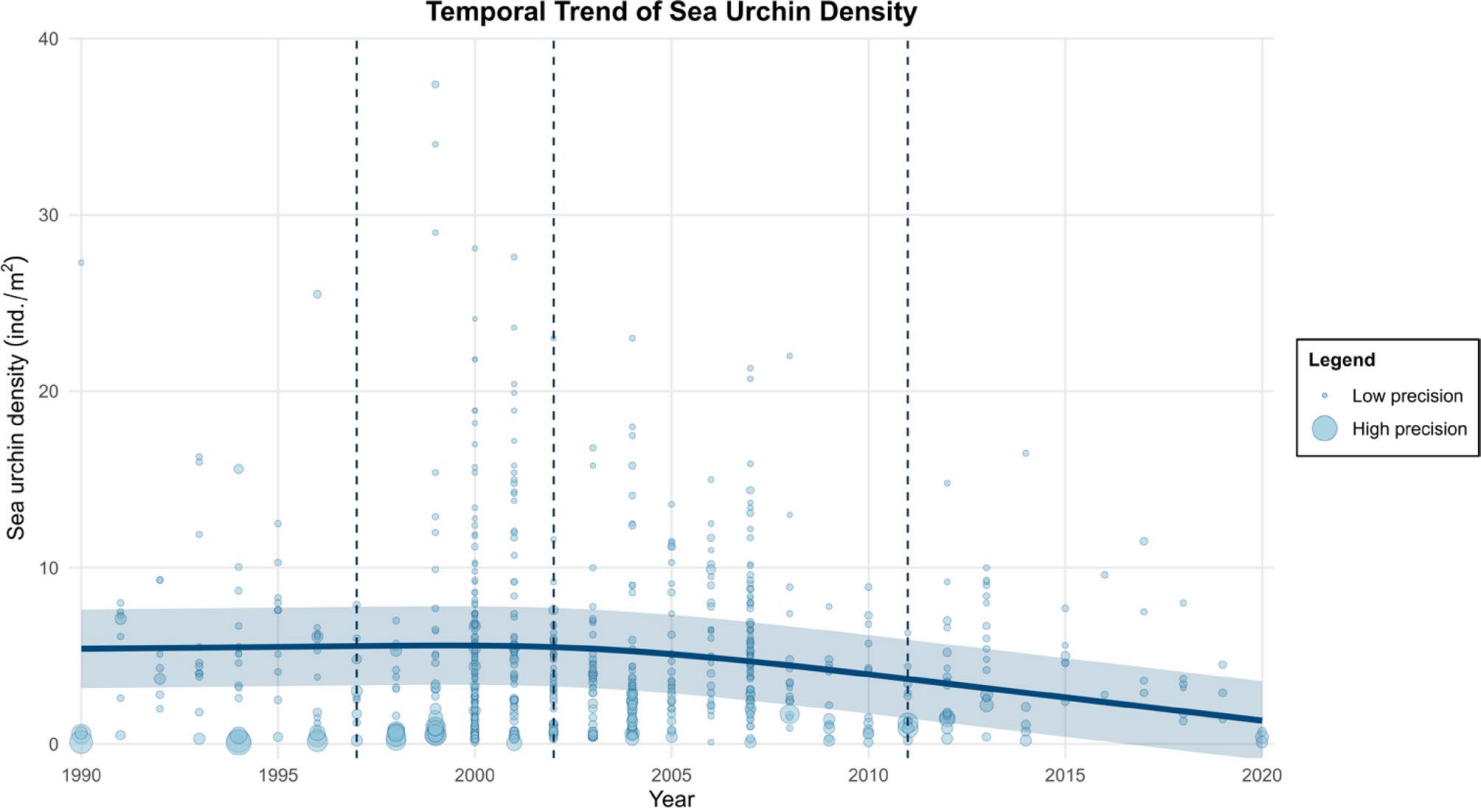
Temporal trend in *Paracentrotus lividus* density across the Mediterranean Sea from 1990 to 2020, based on a meta-analysis of published data. Point size reflects data precision (larger points = large variance). The solid dark blue line represents the estimated trend over time. The shaded area represents the 95% confidence interval, indicating the uncertainty around the estimated trend.. Dashed vertical lines mark the spline knots used in the model.

For the historical analysis of the *P. lividus* densities in the Mediterranean Sea, n. 44 relevant research articles were used. These articles were selected by sorting all articles according to a scale ranging from high relevance to low relevance, with 724 metrics being extracted, representing data on *P. lividus* individual density recorded from 1990 to 2020 from six Mediterranean countries: Spain, France, Italy, Croatia, Greece and Cyprus (Fig. 4). The graphic representation of the 30-year trend of density obtained by this meta-analysis is reported in Fig. 5. We fitted a mixed-effects meta-regression model with random intercepts for “id_work” and “country”, and a restricted cubic spline (RCS), with three knots being applied to the “year” moderator.

The model results indicated a statistically significant relationship between the year and the sea urchin individual density; in fact, the test for moderators gave significant results (${\text{Q}}_{\text{M}}$ (df = 2) = 1814, *p* < 0.0001).

Specifically, the RCS model revealed a non-linear association between year and individual density, with notable changes being observed around the specified knots. The meta-analysis revealed that there is a negative trend in the form of a reduction over time of the average individual density of *P. lividus* in the Mediterranean Sea, which first became manifest in 2003, and which intensified in the following years.

## Discussion

This study documents a dramatic and ongoing decline in *Paracentrotus lividus* populations across the central Mediterranean Sea, based both on a systematic literature review (1990–2020) and on new field surveys conducted in Apulia and in Sicily during summer 2023. Results revealed extremely low *P. lividus* densities (0.2 ind/m^2^), with no significant differences in density values between Marine Protected Areas (MPAs) and non-protected sites. Only 6% of sea urchins sampled in Apulia were of a commercial size, indicating a reduced fecundity and reproductive potential. The literature meta-analysis revealed a clear negative trend in *P. lividus* densities over the past 30 years across the Mediterranean basin. This decline can be attributed to multiple synergistic stressors, including overfishing, sea warming, natural predation, and severe storm events.

The sea urchin *Paracentrotus lividus* is a valuable seafood resource in Italy as in many other Mediterranean countries. The sea urchin roe is an appreciated delicacy and due to an escalating market demand, its small-scale fishery increased over the last two decades across the whole Mediterranean basin^10^, leading to a progressive depletion of the resource.

In Italy, an alarming decline in *P. lividus* populations was detected around the coastal zone of Apulia, Sicily and Sardinia^9^. The results of our recent surveys in summer 2023 demonstrated that sea urchin stocks in Apulia and Sicily nearly collapsed.

This evidence supported the decision of the regional government of Apulia to ban the *P. lividus* fishery up to spring 2026. However, despite the fishery prohibition, sea urchins can be still sold as fresh food in fish markets and restaurants in Apulia if not fished from waters of the same Italian region. They can be imported in Apulia from other Italian regions or from neighboring Mediterranean countries such as Croatia, Albania or Montenegro, where different fishery regulations are implemented. At the same time, an illegal fishery is continuously operating along the Apulia coast, despite strict control actions by the Italian coastguard, especially within MPAs. Similarly, in summer 2023, the Maltese Environment and Resources Authority, through Legal Notice 149/2023, established a 2-year moratorium on the fishery of sea-urchins in Maltese waters, where the density of *P. lividus* had plummeted to 0.004 ± 0.004 (SD) ind m^2^ (AD personal observation). Earlier in 2022, Türkiye stopped the harvesting of this species in the sea of Marmara^52^.

Laboratory experiments showed that the average annual growth rate of *P. lividus* is ~ 17 mm in a controlled condition^53^, while in the field the growth rate seems to be lower, around ~ 10 mm per year^54^. However, these values remain mainly influenced by age (juveniles grow much faster than adults), water temperature, and food availability^55,56^. Apparently, the minimal commercial size (test diameter = 50 mm, without spines) of *P. lividus* is reached in 3–3.5 years in farmed conditions, and in 5–6 years in natural habitats^53,55^. Therefore, the 3-yrs harvesting moratorium introduced in the Apulia region might not be sufficient for the recovery of an exploitable sea urchin population. Further, the number of larger sea urchin specimens (i.e., those with the highest reproductive potential) will decrease drastically when fishery will be re-opened, as observed along north-western Iberian peninsula^57^, thus undoing the re-population scope of the moratorium.

Through this work, we documented the fact that *P. lividus* specimens of commercial test size (≥ 50 mm) constituted just 6% of the total sea urchin specimens sampled along the Apulian coast, from the Tremiti Islands to the Gulf of Taranto (encompassing Southern Adriatic Sea, Otranto Channel, and Northern Ionian Sea). In 2003, the same parameter was approximately 15% in both the MPA and adjacent non-protected area of Porto Cesareo^58^. The density of *P. lividus* individuals of marketable test size is influenced by the success of recruitment which, in turn, depends on the presence of breeding individuals^59^. These specimens are usually the most exposed ones to harvesting, which therefore reduces the overall reproductive potential of populations^15^. At the same time, larval connectivity plays a crucial role in the distribution of sea urchin individuals^18^.

In Italy, human exploitation appears to be the main driver of the observed decline of *P. lividus* populations^7,9,60–63^. The comparative analysis of data retrieved from MPAs and non-protected areas around the coast of Apulia and Sicily revealed no significant differences in the distribution of *P. lividus*. The density detected in Apulia inside the MPA of Torre Guaceto ranged from 0.3 to 1.1 ind/m^2^ (in 2002^64^) to 0.1 ind/m^2^ (in 2023; present work) over a period of almost 20 years. The same phenomenon was observed also in non-protected area, where the *P. lividus* density decreased more drastically over the same period, from 1.2 to 7.6 ind/m^2^ in 2002^64^ to 0.2 ind/m^2^ in 2023 (present work). In Sicily, we detected a highly comparable negative trend in *P. lividus* individual density values. In the MPA of the island of Ustica, for instance, the density decreased over time, from 1.5 to 4 ind/m^2^ in 2003 to 1.1 ind/m^2^ in 2023^7^ (present work).

A similar negative trend over time was reported by Coppa et al.^9^ for the Sardinia region, where the average density of sea urchins decreased at similar rates for MPAs and non-protected sites, with a strong reduction (− 73%) in sea urchin stock, over a 9-year period (from 2010 to 2018).

MPAs function as conservation areas for numerous fish species^65^, particularly those that prey on sea urchins (e.g., several representatives of Sparidae). Therefore, MPAs do not act as recovery areas for sea urchins, these being controlled by natural predators^28^. Conversely, in non-protected areas, although the occurrence of fish predators is reduced by fishing activities, human predation on *P. lividus* (when and where allowed) may act as a key factor controlling the decline of sea urchin populations in the central Mediterranean Sea; this hypothesis is supported by the fact that, within the Torre Guaceto MPA (south–east Italy), recorded fish abundance and size values are larger with respect to corresponding values for the non-protected areas^66^. However, Historical fishing pressure within MPAs, prior to their establishment or due to lax enforcement, may also contribute to the lack of density differences in the past.

Indeed, besides natural predation and overfishing, the abundance and distribution of *P. lividus* individuals seem to be influenced by global warming and storm activity. Sea surface temperature (SST) is considered a critical factor that has increasingly reduced *P. lividus* individual abundance in the Levantine Sea, where the average SST is much higher than in other regions of the Mediterranean Sea^36,40,67^. Despite this, so far, there is no documented evidence of large-scale mortality of *P. lividus* linked to marine heat waves (MHWs). Only two mass mortalities of sea urchins have been reported in the Mediterranean Sea: in the coastal areas of Port Cros (France) in 1980, due to a bacterial disease (the so-called bald-sea-urchin disease)^68,69^ and in the Medes Island (Spain), after a severe storm hit in 2008^70,71^. However, an impressive decreasing trend has been documented in Israel, where *P. lividus* density ranged 2–6 ind/m^2^ in 1994^36^, but was found to be extremely rare in 2014–2015 all along the Israeli coast^40^. A few years later, in 2021–2022, no *P. lividus* were recorded along the entire coast of Israeli^72^ as well as from the southern Lebanese waters (Tyre Natural Reserve; AT, personal observation). In addition, *P. lividus*, appears to be rapidly disappearing coastal waters off Crete and Cyprus, and generally from the South Aegean Sea, whereas its populations are stable and healthy in the northern Aegean Sea^72^.

In Türkiye, in 2002, *P. lividus* individual densities were high, reaching 12 ind/m^2^^37^ and in Egypt, for the years 2014–2015, the individual density of *P. lividus* was also very high, up to 50 ind/m^2^^38^. A few years later, the calculated fishing mortality of *P. lividus* near Alexandria contributed to almost 73% of its total mortality^47^, documenting the heavy exploitation of local sea urchin stocks and supporting an urgent adoption in Egypt of *P. lividus* fishery management actions, such as the introduction of daily harvesting quotas, a minimum harvestable size of 45 mm, a prolonged no-take period (from December to May) and the establishment of no-take refuge zones^47^.

Overall, SST and overfishing can be considered as key factors in driving the collapse of *P. lividus* populations in the warmer Levantine Sea; besides, additive temperature-related mechanisms should be considered, such as the increase on natural mortality rates due to the arrival of non-indigenous (NIS) sea urchin predators in the Mediterranean through the Suez Canal^47^ (the so-called ‘Lessepsian migrants’) or the reduced viability of sea urchin stock populations due to resource competition by highly motile NIS herbivore species, such as fish^68^ or invertebrates^73^.

Overall, our systematic literature review highlighted a decreasing trend in the individual density of *P. lividus* for the entire Mediterranean Sea, extending over the last three decades (1990–2020), suggesting that multiple interacting factors, including overfishing, rising sea temperatures, biological invasions, and extreme weather events, are synergistically driving the observed long-term ongoing decline at basin scale. The result of meta-analysis suggests that, in the Mediterranean Sea, the decreasing trend in *P. lividus* started as of 2003. Notably, this temporal onset coincides with one of the most intense and prolonged marine heatwaves (MHWs) ever documented in the region in summer 2003^74^.

Following this MHW, the Mediterranean Sea, particularly the Levantine Basin, has been subjected to several successive marine heatwaves (MHWs), occurring both in summer and in winter during the last 2 decades^75^. Severe mass mortality was observed after the MHW of 2003, particularly affecting corals and sponges throughout the Mediterranean Sea^76^.At basin level, we hypothesize that this decreasing trend is being driven by multiple factors, each locally exerting a specific impact, according to the geographical region, the spatial and temporal extent of harvesting and the absence or presence of fishery regulations. In the Levantine Sea, we consider high sea surface temperatures (SST), along with the presence of non-indigenous predators and competitors, to be the main drivers of sea urchin decline; in the central Mediterranean Sea, the main impact on the sea urchin stock viability is presumably related to overfishing, as there are currently no studies directly linking sea temperatures to the decline of *P. lividus* populations in this area of the basin. In the north-western Mediterranean Sea, there is limited evidence of population decline, with the available data pointing mainly to episodic events such as severe storms^69^ and natural predation by fish as the primary factors influencing *P. lividus* density.

All these factors, acting at various levels across different regions, can significantly impact on the abundance of *P. lividus* in the Mediterranean Sea. This highlights the importance of managing edible sea urchin populations through the adoption of different approaches, optimized on a sub-regional basis, depending on the most important pressure acting on local sea urchin stocks.

The current legislation on the fishery of *P. lividus* in Italy (MIPAAF, Ministerial Decree 12 January 1995: daily quotas up to 1000 individuals for professional fishermen; minimum harvestable size 7 cm, including spines; no take periods in May and June) does not seem sufficient to guarantee the sustainability of its exploitation. The regulation is based on old scientific information, dated more than 30 years ago. The daily quotas should be better modulated on the carrying capacity and distribution of this species, i.e., based on a priori assessment of the wild stocks and on the local reproductive periods of the species. Moreover, in Italy the current harvesting limit of 1000 sea urchins per day per professional fisher, with no weekly restriction, is unsustainable. A more effective approach would be to limit harvesting to 500 specimens per day, for no more than three days per week, allowing each fisher to collect a maximum of 1500 sea urchins per week instead of the current 7000 specimens. Recreational harvesting should be abolished, as it is currently impossible to obtain a reliable estimate of the number of individuals removed each year through this practice.

In addition, the current biological closure period for *P. lividus* in Italy takes place in May and June. This regulation should be revised, as reproductive activity in *P. lividus* shows multiple peaks throughout the year, typically in August, February, March, and April, depending on environmental conditions^77^. Reproductive activity in May is generally minimal, as confirmed by low gonadosomatic index (GSI) values in the Northern Ionian Sea^77^. Therefore, the current closure period, which includes May and June, does not coincide with the most critical reproductive phases. Adjusting the current closure window to better align with actual reproductive peaks would improve the protection of spawning individuals and enhance recruitment success.

Beyond regulatory measures, alternative strategies such as aquaculture and restocking programs could play a complementary role in ensuring the sustainability of *P. lividus* populations. Controlled aquaculture could help reduce the pressure on wild stocks while maintaining market availability, as successfully demonstrated in other fisheries. Additionally, restocking efforts could be explored in areas where populations have severely declined, with careful management to avoid ecological imbalances. However, these approaches present challenges, including potential genetic risks, competition with wild populations, and the need for cost-effective farming techniques. Future research should assess the feasibility of these solutions in a Mediterranean context. At the same time, based on the data presented in this study, areas where sea urchin densities were found to be completely absent should be permanently closed to fishing. These areas could act as natural refuges, allowing for potential recolonization if environmental conditions permit. They may also become suitable sites for passive restocking initiatives, enabling an evaluation of the ecological outcomes of reintroduction programs.

A total fishing ban could have significant economic consequences for local fishers, particularly those who rely on *P. lividus* harvesting as a primary source of income. The lack of alternative livelihoods could lead to financial instability, increased illegal harvesting, or a shift to other marine resources, potentially creating new ecological pressures. At the same time, estimating the actual resultant economic loss is extremely difficult, as most fishers do not formally declare how much they actually harvest, nor how much they are losing due to the ban. This lack of transparency represents a major challenge for fisheries management. Nonetheless, The Apulia Region has recently allocated financial support to sea urchin fishers whose activities have been officially suspended following the implementation of the three-year moratorium on sea urchin harvesting.

For example, in the entire region of Sicily (1520 km of coastline), only twelve fishers are officially registered to harvest sea urchins legally, despite evidence that sea urchins are sold daily in restaurants and fish markets throughout the region.

Previous studies revealed, as one of the effects of the severe harvesting of *P. lividus*, the increasing growth rate, size, and abundance of the non-harvested sea urchin *Arbacia lixula* associated with the barren formation^78^, resulting in a consequent loss of biodiversity^79^, and in the collapse of ecosystem functions^80^. This situation should motivate the formulation of new management plans and protocols to restore declining production of the purple sea urchin.

To assess the overall health status of the purple sea urchin in the Mediterranean basin and to delineate coordinated transnational action plans, it will be essential to gather information on *P. lividus* population densities across the entire region, particularly along the southern borders of the Mediterranean. Currently, available data on the purple sea urchin from North African coasts are limited to its reproductive biology, biometric measurements, and the use of sea urchins as ecological bioindicators^81–84^, highlighting the need for more comprehensive studies on the populations health status and on the available genetic resources at basin scale.

In conclusion, it appears that the persistent decline of *P. lividus* in the Mediterranean Sea will require context-specific, adaptive management strategies, and transnational coordination. Without effective regulations and monitoring, the recovery of depleted populations remains unlikely. Further research is needed to clarify the relative contribution of each stressor and to support evidence-based conservation actions. Long-term monitoring and improved enforcement are essential to locally restore population structure and reproductive capacity. Urgent actions at national and transnational levels are crucial to reduce the synergistic impacts of multiple anthropogenic causes and avoid future eradication of an ecologically and economically important species.

## Methods

### Study area and survey: Apulia

**Fig. 6:**
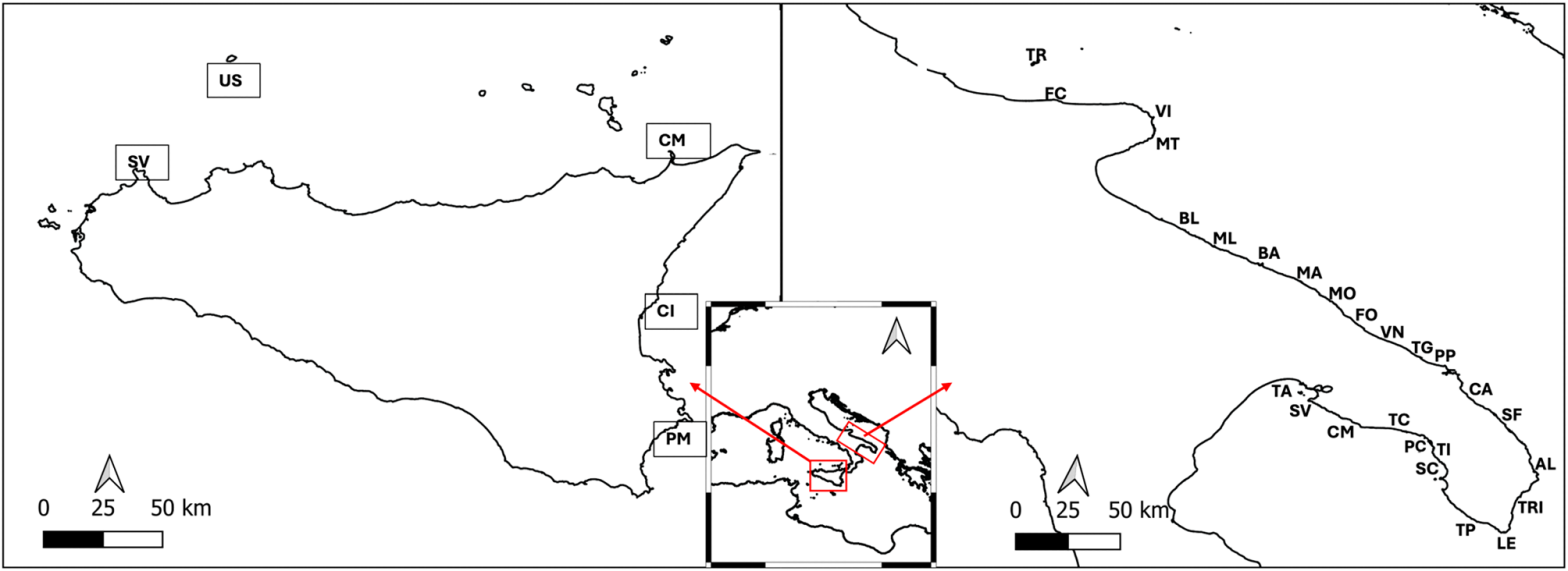
Map of the study site in Italy. TR, Tremiti Islands (MPA); FC, Varano; VI, Vieste; MT, Mattinata; BL, Barletta; ML, Molfetta; BA, Bari; MA, Mola di Bari; MO, Monopoli; FO, Forcatella; VN, Villanova; TG, Torre Guaceto (MPA); PP, Punta Penne; CA, Casalabate; SF, San Foca; AL, Alimini; TRI, Tricase Porto; LE, Santa Maria di Leuca; TP, Torre Pali; SC, Santa Caterina; TI, Torre Inserraglio; PC, Porto Cesareo (MPA); TC, Torre Colimena; CM, Campomarino; SV, San Vito; TA, Taranto; CM, Capo Milazzo; US, Ustica; SV, San Vito; PM, Plemmirio; CI, Ciclopi Island.

In summer 2023, a field survey was carried out along the coastal rocky bottoms of the Apulia region. N. 26 sites were randomly selected along the coastal zone, avoiding sandy patches (Fig. 6).

Three of these sites were located inside Marine Protected Areas (InMPAs), where harvesting of marine organisms is completely forbidden. The survey was carried out on rocky bottoms colonized by photophilic algae as well as by scattered patches of *Posidonia oceanica*.

At each site, the same underwater sampling protocol was replicated three times, extending in a shore-normal direction (perpendicularly to the coastline) over a maximum linear length of 100 m or down to a maximum depth of 10 m. The underwater survey was conducted by two scientific SCUBA divers, who recorded the occurrence of *P. lividus* specimens within a 3 m-wide corridor to the right and to the left of the measuring tape which had previously been stretched out on the seabed, thus effectively implementing a belt transect approach. The overall monitoring effort resulted in a whole survey area of 600 m^2^ per replicate transect, i.e., a total survey of 1800 m^2^ per site. All the sea urchins in the corridor were counted and measured with a vernier caliper, measuring the test diameter without the spines. Then, the acquired data were sorted into 7 size classes: (I) 0–10 mm; (II) 10–20 mm; (III) 20–30 mm; (IV) 30–40 mm; (V) 40–50 mm; (VI) 50–60 mm; (VII) > 60 mm.

Permutational multivariate analyses of variance (two-way PERMANOVA) were used to detect any significant differences between samples from protected and non-protected areas (fixed factor), in the relative abundance of urchin size classes and among sites (nested random factor). The Zero-adjusted Bray–Curtis distance was applied so as to construct the similarity matrix, in order to factor in any potential distortions arising from the relatively high number of sites with no sea urchins^85,86^. Secondly, the difference in the total density of sea urchins between the two types of sampling sites was also assessed through the same design, but the similarity matrix was computed using Euclidean distance in this case.

### Study area and survey: Sicily

In the summer 2023, a second sampling survey was also carried out in Sicily, in order to detect the individual density of *P. lividus* inside and outside five different marine protected areas (Inside and non-protected MPAs).

The survey was carried out within a consistent depth range of 0–15 m across all sites, focusing on two distinct habitats: (i) photophilic algae (0–10 m), with 10 transects (5 inside MPAs and 5 in non-protected areas), and (ii) *Posidonia oceanica* meadows (10–15 m), with 10 transects (5 inside MPAs and 5 in non-protected areas) (Fig. 6).

For each transect, having a maximum length of 100 m, 10 replicates were deployed, parallel to the coast, where the presence and the size of sea urchins were investigated by placing a 1 × 1 m quadrat every 10 m along the same transect. All the sea urchins found were measured with a vernier caliper, but, in this second survey, the measurements include the spines’ length. In this case, permutational multivariate analysis of variance (two-way PERMANOVA) was used in order to detect any significant differences between samples from protected and non-protected areas (fixed factor) in the relative abundance of urchin size classes and among sites (crossed random factor) with protection. The Zero-adjusted Bray–Curtis distance was applied in order to construct the similarity matrix, in order to factor in any potential distortions arising from the relatively high number of sites with no sea urchins^85,86^. Secondly, the difference in the total individual density of sea urchins between the two types of sites was assessed with the same design, but the similarity matrix was computed using Euclidean distance in this case. All the statistical analyses were performed only for the photophilic algae assemblage, given that, within the *Posidonia oceanica* meadows, just one *P. lividus* individual was recovered from all the sampled locations. All the statistical analyses on the datasets from Apulia and Sicily were carried out using the software PRIMER v. 6 including the PERMANOVA add-on package^87,88^.

### Historical analysis (1990–2020) of *P. lividus* density across the Mediterranean Sea

In order to infer trends in the individual density of *P. lividus* across the Mediterranean Sea, scientific papers published over the period 1990–2020 were retrieved from Scopus and Google Scholar databases, applying the joint keywords “*Paracentrotus lividus*” and “Mediterranean Sea” and “density”. The retrieved articles were cross-checked for datasets on *P. lividus* average individual density (± standard deviation or standard error) and the number of applied sampling replicates. Numerical data were also gathered from graphs in the published articles, using the online version of “Plot Digitizer”^89^. Articles reporting sea urchin densities equal to zero were excluded from the current analysis in order to avoid introducing bias from areas where the species has never been present. Finally, any article reporting a mean individual density value calculated over different years but not supported by annual monitoring was not included in the present analysis.

The final retrieved dataset was analyzed using the “metafor” package (Version 4.6.0) and the ‘rms’ package (Version 6.8.1) in R (Version 4.4.1). A meta-regression was performed in order to assess the overall effect of variation in the density of *P. lividus* over 30 years. In particular, a mixed-effect restricted cubic spline model was used to handle non-linear relationships between variables. The model accounts for the identification number (“id_work”) and the different country within the study as random factors to address heterogeneity, and the year of sampling as a fixed factor^90^. An omnibus test (${\text{Q}}_{\text{M}}$ test statistic) of the moderator (fixed factor) was then conducted to assess the overall significance of the fixed effect. The effect exerted by the publication year on the outcome was modeled using restricted cubic splines (RCS) so as to capture potential non-linear relationships. The selection of knots for the spline model followed Harrell’s recommended percentiles^91^ approach, with three knots positioned at the 10th (1997), 50th (2003), and 90th (2012) percentiles for the variable ‘year’, allowing the model to flexibly adapt to changes over time. This method ensures an optimal balance between model complexity and interpretability, avoiding overfitting while preserving the flexibility to detect non-linear trends. In this study, the random effects were incorporated as hierarchical random intercepts for both study “Id_work” and “country”, capturing potential clustering effects and unmeasured variability between studies.

## Electronic supplementary material

Below is the link to the electronic supplementary material.

Supplementary Material 1

## Data Availability

In Supplementary Material 1, the list of references used for data collection in the meta-analysis is provided. The dataset is available from the corresponding author (AT) on request.
